# Supplementary material for: Bak and Bcl-xL Participate in Regulating Sensitivity of Solid Tumor Derived Cell Lines to Mcl-1 Inhibitors
Source: Cancers (Basel). 2021 Dec 30;14(1):181. doi: 10.3390/cancers14010181 (PMC8750033; doi:10.3390/cancers14010181)
Supplement: Supplementary file 1 [file cancers-14-00181-s001.zip › cancers-1419350-supplementary.pdf]

cancers

MDPI

Supplementary data

Bak and Bcl-xL participate in regulating sensitivity of solid-tumor derived cell lines to Mcl-1 inhibitors

Viacheslav V. Senichkin †, Nikolay V. Pervushin †, Alexey V. Zamaraev, Elena V. Sazonova, Anton P. Zuev, Alena Y. Streletskaia, Tatiana A. Prikazchikova, Timofei S. Zatsepin, Olga V. Kovaleva, Elena M. Tchevkina, Boris Zhivotovsky* and Gelina S. Kopeina *

Table S1. Sequences of siRNAs

Bak siRNAs (gene: *BAK1*)		
#1	Sense	5′-GuAcGAAGAuucuucAAAuTsT-3′
	Antisense	5′-AUUUGAAGAAUCUUCGuACTsT-3′
#2	Sense	5′-ucAucGGGGAcGAcAucAATsT-3′
	Antisense	5′-UUGAUGUCGUCCCCGAUGATsT-3′
#3	Sense	5′-GccuAuGAGuAcuucAccATsT-3′
	Antisense	5′-UGGUGAAGuACUcAuAGGCTsT-3′
#4	Sense	5′-GcuucGuGGucGAcuucAuTsT-3′
	Antisense	5′-AUGAAGUCGACcACGAAGCTsT-3′
#5	Sense	5′-uGAcccGcuucGuGGucGATsT-3′
	Antisense	5′-UCGACcACGAAGCGGGUcATsT-3′
#6	Sense	5′-AAccGAcGcuAuGAcucAGAGTsT-3′
	antisense	5′-CUCUGAGUcAuAGCGUCGGUUTsT-3′
#7	sense	5′-AAGcGAAGucuuuGccuucTsT-3′
	antisense	5′-GAAGGcAAAGACUUCGCUUTsT-3′

Uppercase letters: ribonucleotides, lowercase letters: 2’-O-Methyl nucleotides, s: phosphorothioate.

Bax siRNAs (gene: *BAX*)		
#1	Sense	5′-uuuucuGAcGGcAAcuucATsT-3′
	antisense	5′-UGAAGUUGCCGUcAGAAAATsT-3′
#2	sense	5′-GGAAcuGAucAGAAccAucTsT-3′
	antisense	5′-GAuGGUUCUGAUcAGUUCCTsT-3′
#3	sense	5′-GccGGGuuGucGcccuuuuTsT-3′
	antisense	5′-AAAAGGGCGAcAACCCGGCTsT-3′
#4	sense	5′-GGAuGAuuGccGccGuGGATsT-3′
	antisense	5′-UCcACGGCGGcAAUcAUCCTsT-3′
#5	sense	5′-AAcuGAucAGAAccAucAuTsT-3′
	antisense	5′-AUGAUGGUUCUGAUcAGUUTsT-3′

Uppercase letters: ribonucleotides, lowercase letters: 2′-O-Methyl nucleotides, s: phosphorothioate.

**Bim siRNAs (gene: *BCL2L11*)**		
#1	sense	5′-ccuuAuGAGuGcAGuGGAATsT-3′
	antisense	5′-UUCcACUGcACUcAuAAGGTsT-3′
#2	sense	5′-AAuGGAAGuuuGuuGuGAATsT-3′
	antisense	5′-UUcAcAAcAAACUUCcAUUTsT-3′
#3	sense	5′-uAuAcuGAuccAucAcuAATsT-3′
	antisense	5′-UuAGUGAUGGAUcAGuAuATsT-3′
#4	sense	5′-ucGAGcGAucuGuuAuuAATsT-3′
	antisense	5′-UuAAuAAcAGAUCGCUCGATsT-3′
#5	sense	5′-AcGAcuGuuAcGuuAcAuuTsT-3′
	antisense	5′-AAUGuAACGuAAcAGUCGUTsT-3′
#6	sense	5′-AccAcccAcGAAuGGuuAuTsT-3′
	antisense	5′-AuAACcAUUCGUGGGUGGUTsT-3′
#7	sense	5′-AuuGcAGccuGcGGAGAGGTsT-3′
	antisense	5′-CCUCUCCGcAGGCUGcAAUTsT-3′
#8	sense	5′-AccGAGAAGGuAGAcAAuuTsT-3′
	antisense	5′-AAUUGUCuACCUUCUCGGUTsT-3′
#9	sense	5′-GuGAccGAGAAGGuAGAcAAuuGcAGcTsT-3′
	antisense	5′-GCUGcAAUUGUCuACCUUCUCGGUcACTsT-3′
#10	sense	5′-cuAccucccuAcAGAcAGATsT-3′
	antisense	5′-UCUGUCUGuAGGGAGGuAGTsT-3′

Uppercase letters: ribonucleotides, lowercase letters: 2′-O-Methyl nucleotides, s: phosphorothioate.

Figure S1

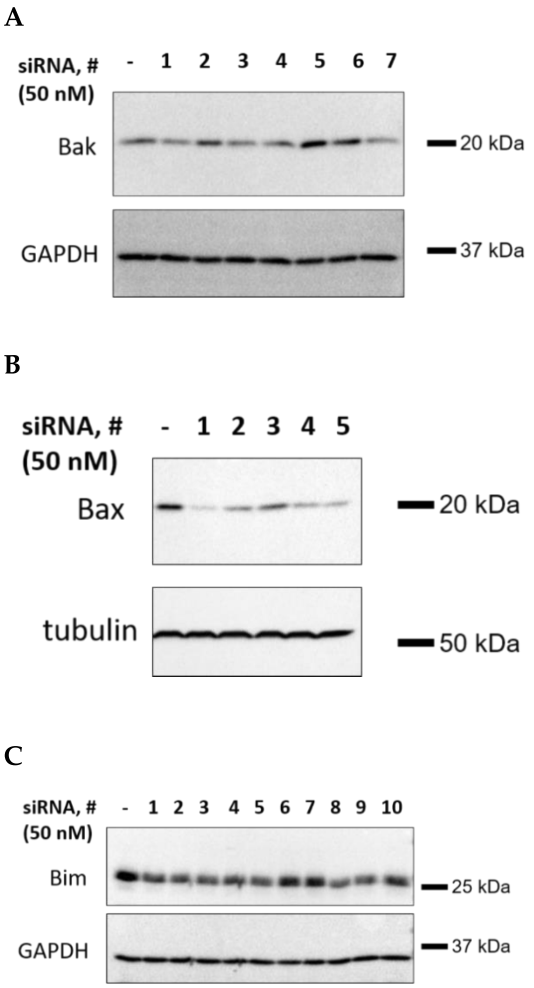

Figure S1. Testing of efficiency of all siRNAs. Cells were untransfected (-) or transfected with siRNAs for *BAK1* (**A**), *BAX* (**B**), or *BCL2L11* (**C**). All siRNAs were used at 50 nM for 24 h. Sequences of each siRNA are represented in Table S1. Tubulin and GAPDH were used as loading controls.

Figure S2

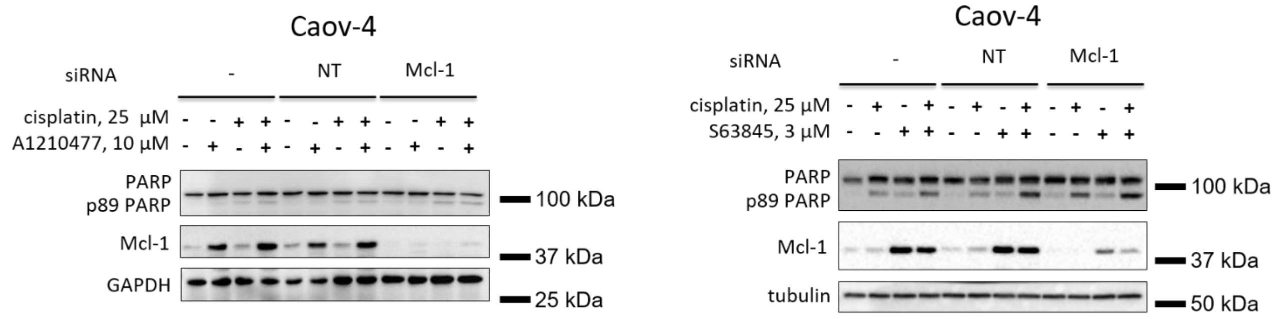

Figure S2. Western blot (WB) analysis of Caov-4 cells treated with 10 µM A1210477 and 3 µM S63845, either alone or in combination with 25 µM cisplatin. Cells were untransfected (-), transfected with non-targeting siRNA (NT) or with siRNA to *MCL1* (Mcl-1). Data from n = 3 biological replicates. Tubulin and GAPDH were used as loading controls. Time of incubation: 24 h, time of transfection – 6 h.

Figure S3

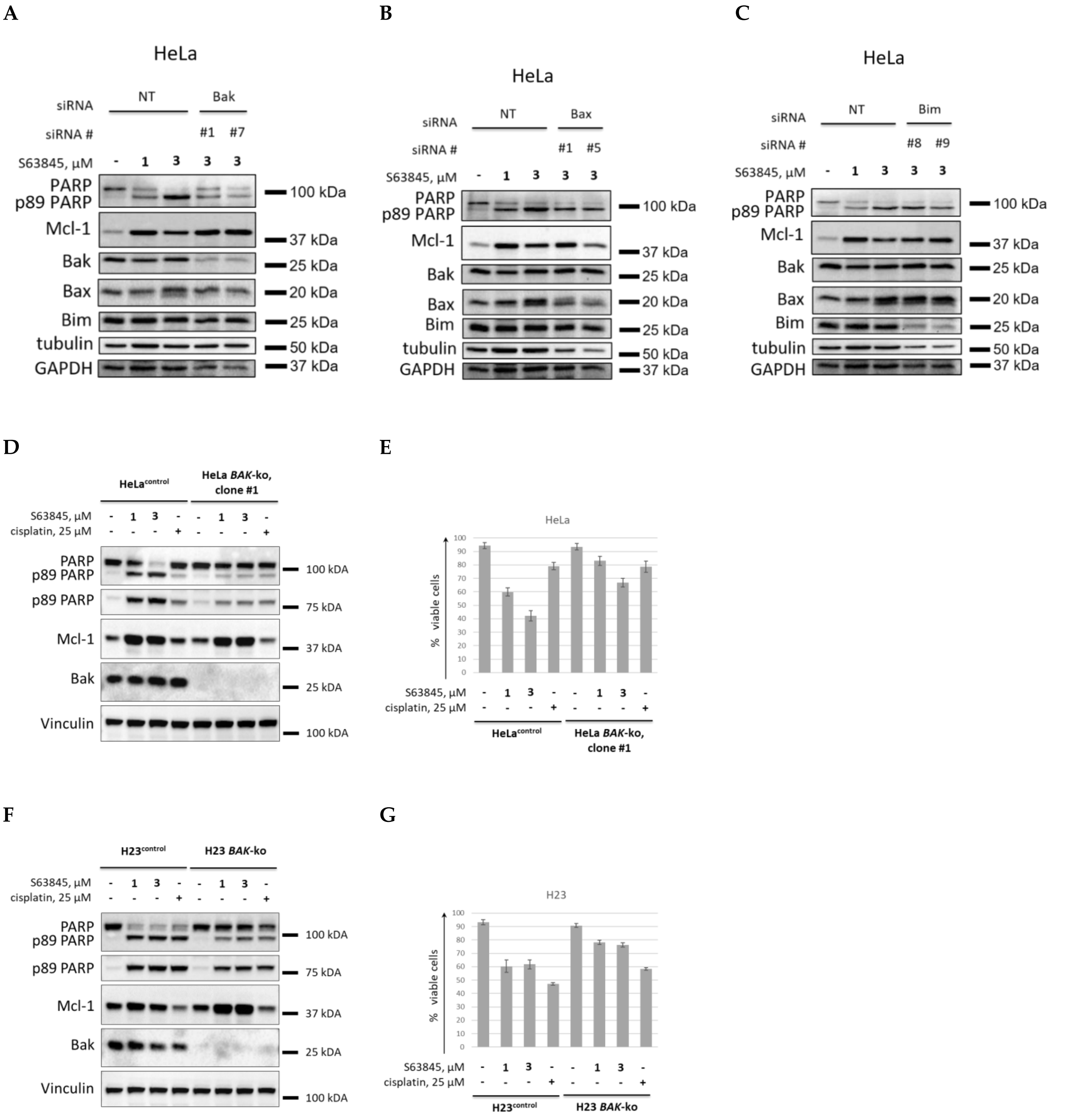

Figure S3. Testing of efficiency of selected siRNAs and analysis of the efficiency of cisplatin in Bak-knockout cells. Cells were untransfected (-) or transfected with siRNAs for *BAK1* (**A**), *BAX* (**B**), or *BCL2L11* (**C**). All siRNAs were used at 50 nM for 24 h. Sequences of each siRNA are represented in Table S1. NT – non-targeting siRNA. Tubulin and GAPDH were used as loading controls. WB (**D**) and FACS analysis (**E**) of HeLa cells with or without knockout of BAK1. Cells were treated with 1 or 3 µM S63845 and 25 µM cisplatin. WB (**F**) and FACS analysis (**G**) of H23 cells with or without knockout of BAK1. Cells were treated as

described in D. Data from $n = 3$ biological replicates are shown as mean ± s.d.; % viable cells – cells negative for both Annexin V-FITC and propidium iodide (PI). Vinculin was used as a loading control. Time of incubation: 24 h.

Figure S4

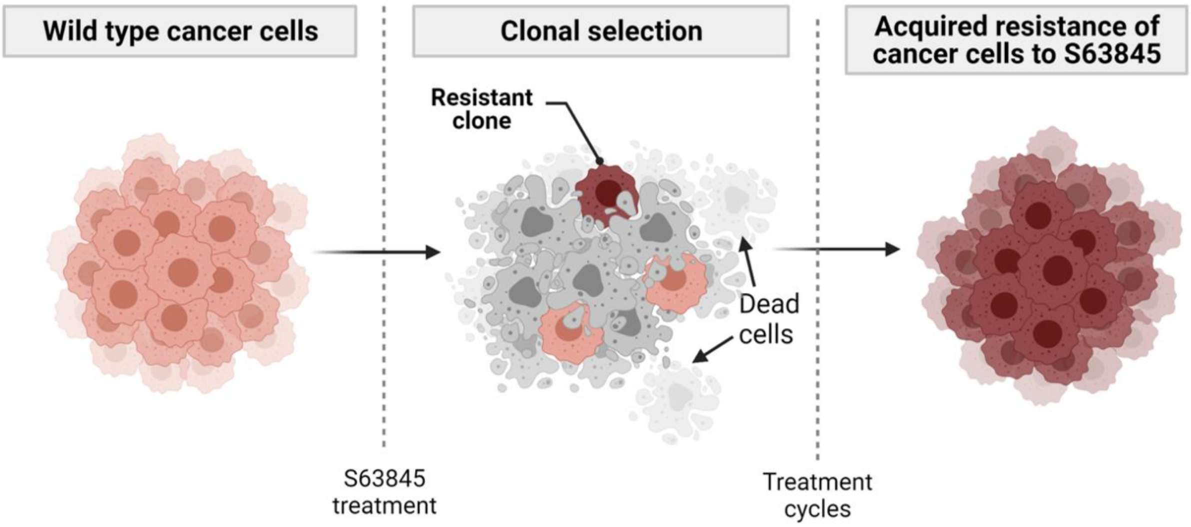

Figure S4. Representation of the principles of raising cells with acquired resistance to S63845. Created with BioRender.com

Figure S5

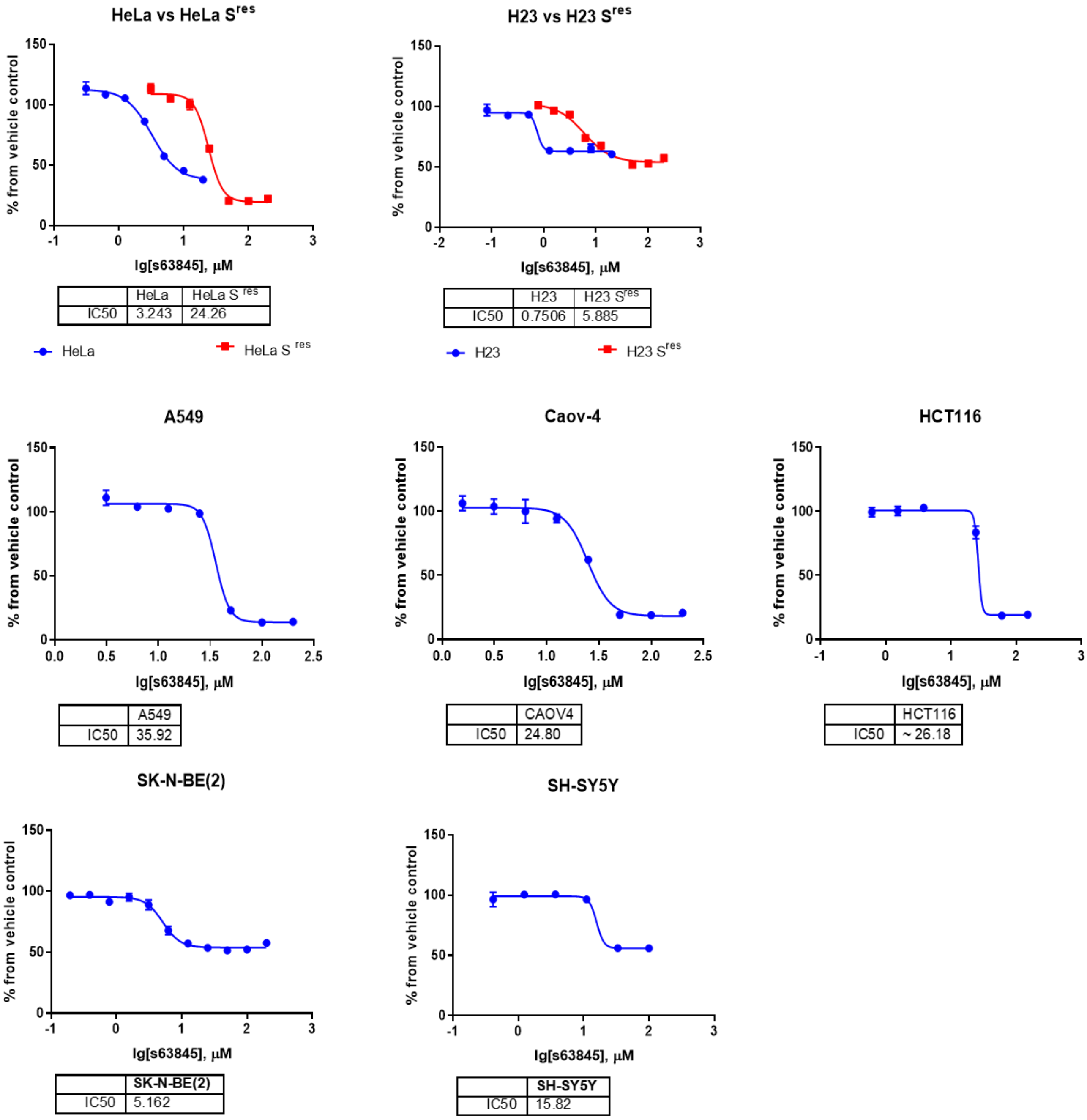

Figure S5. MTS cell viability assay of the cancer cell lines upon treatment with selected concentarions of S63845. Data from n = 3 biological replicates are shown as mean ± s.d. Time of incubation: 24 h.

Figure S6

A

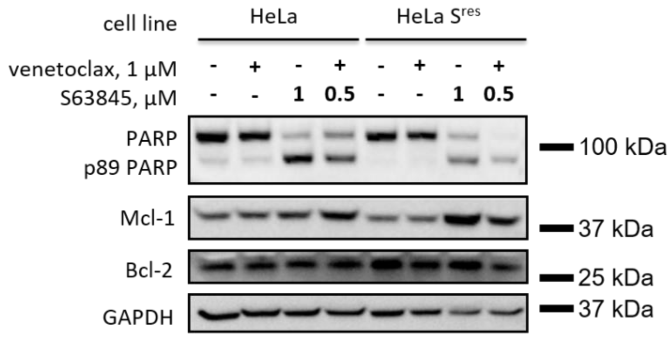

B

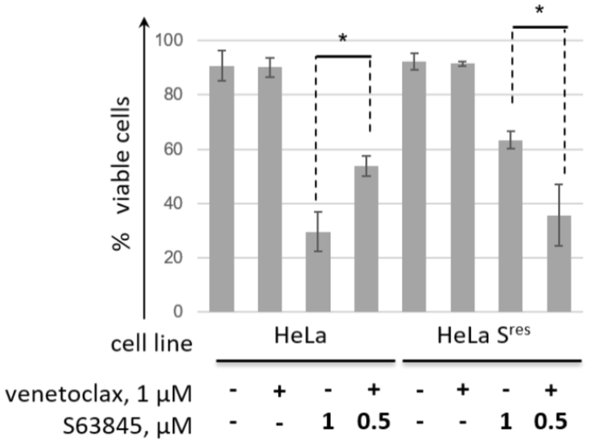

Figure S6. Assessment of the efficiency of concomitant inhibition of Mcl-1 and Bcl-2 in parental HeLa cells and HeLa cells with acquired resistance to S63845 (HeLa S^{res}). WB analysis (**A**) and FACS analysis (**B**) of HeLa and HeLa S^{res} cells treated with S63845 and venetoclax alone (1 µM each) or with their combination (0.5 µM S63845 and 1 µM venetoclax). Data from $n = 3$ biological replicates are shown as mean ± s.d., * $p < 0.05$; % viable cells – cells negative for both Annexin V-FITC and propidium iodide (PI). GAPDH was used as a loading control. Time of incubation: 24 h.

Figure S7

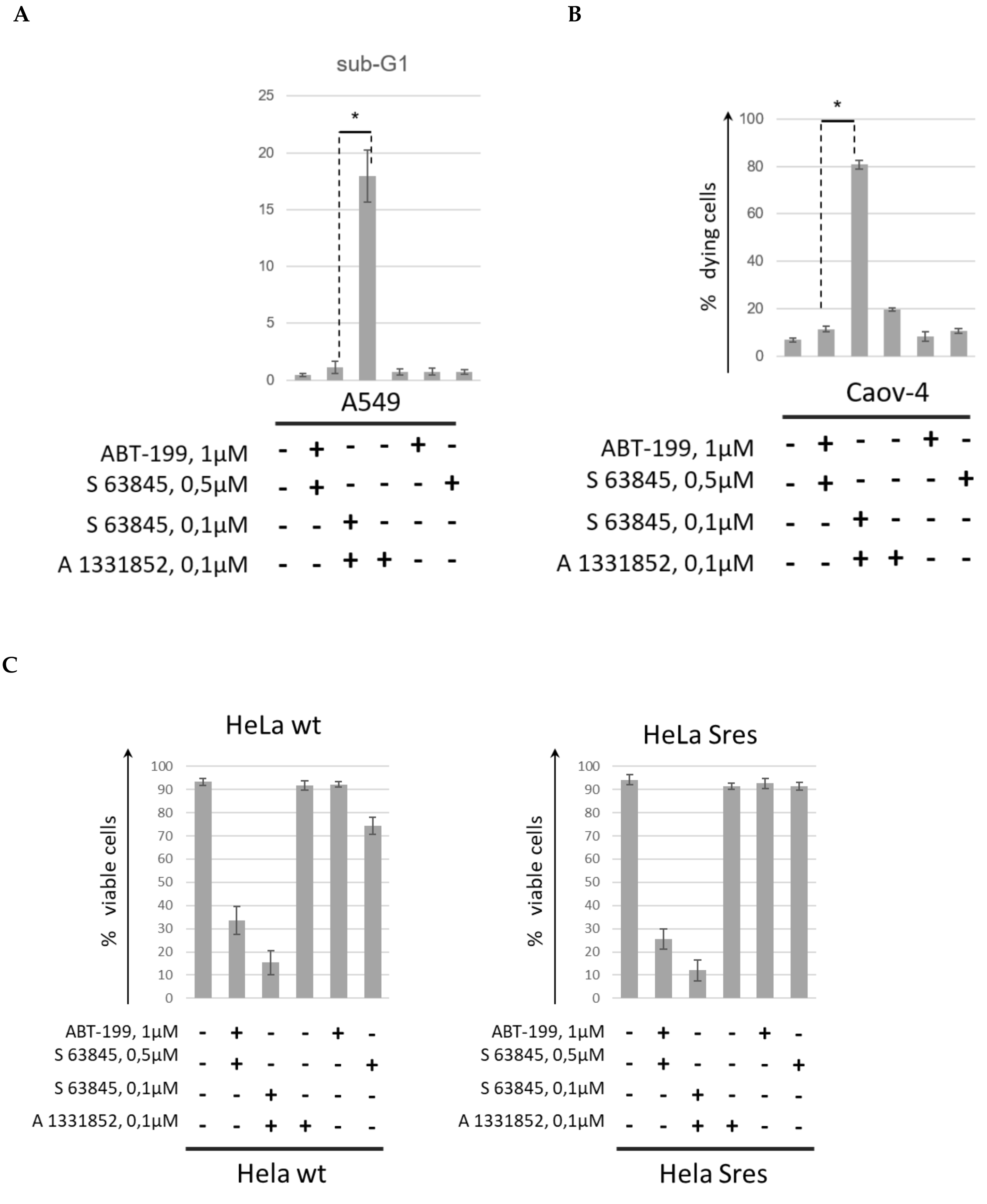

Figure S7. Assessment of the efficiency of combination of S63845 with BH3 mimetics to Bcl-2 (ABT-199, venetoclax) or Bcl-xL (A1331852). FACS analysis of A549 cells using Sub-G1 test (**A**), Caov-4 (**B**), HeLa and HeLa S^{res} cells (**C**) using Annexin V-FITC / PI staining. Data from n = 3 biological replicates are shown as mean ± s.d., * $p < 0.05$. In A, percent of Sub-G1 population is shown. In B, % dying cells – cells positive for Annexin V-FITC and/or propidium iodide (PI). Time of incubation: 6 h.

Figure S8

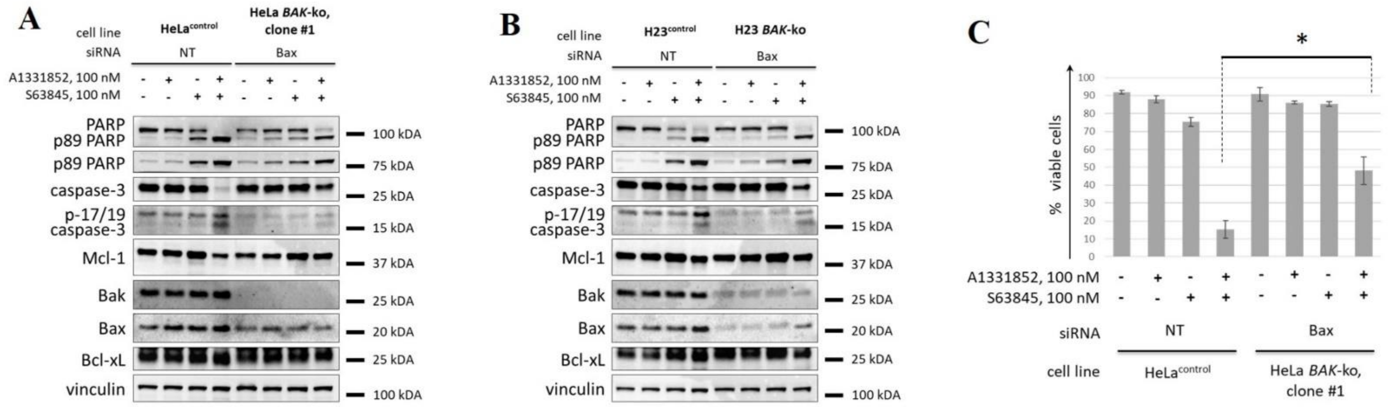

Figure S8. Analysis of the efficiency of concomitant inhibition of Mcl-1 and Bcl-xL in Bak-knockout cells. **A, B.** WB analysis of HeLa (A) or H23 (B) cells with or without knockout of BAK1. Cells were transfected with non-targeting siRNA (NT) or with siRNA BAX (Bax) and treated with 100 nM S63845, 100 nM A1331852, or both. siRNA #1 (see Table S1). **C.** FACS analysis of HeLa cells with or without knockout of BAK1. Cells were treated as described in A. Data from n = 3 biological replicates are shown as mean ± s.d., * $p < 0.05$; % viable cells – cells negative for both Annexin V-FITC and propidium iodide (PI). *Vinculin* was used as a loading control. Time of incubation: 6 h.

Figure S9

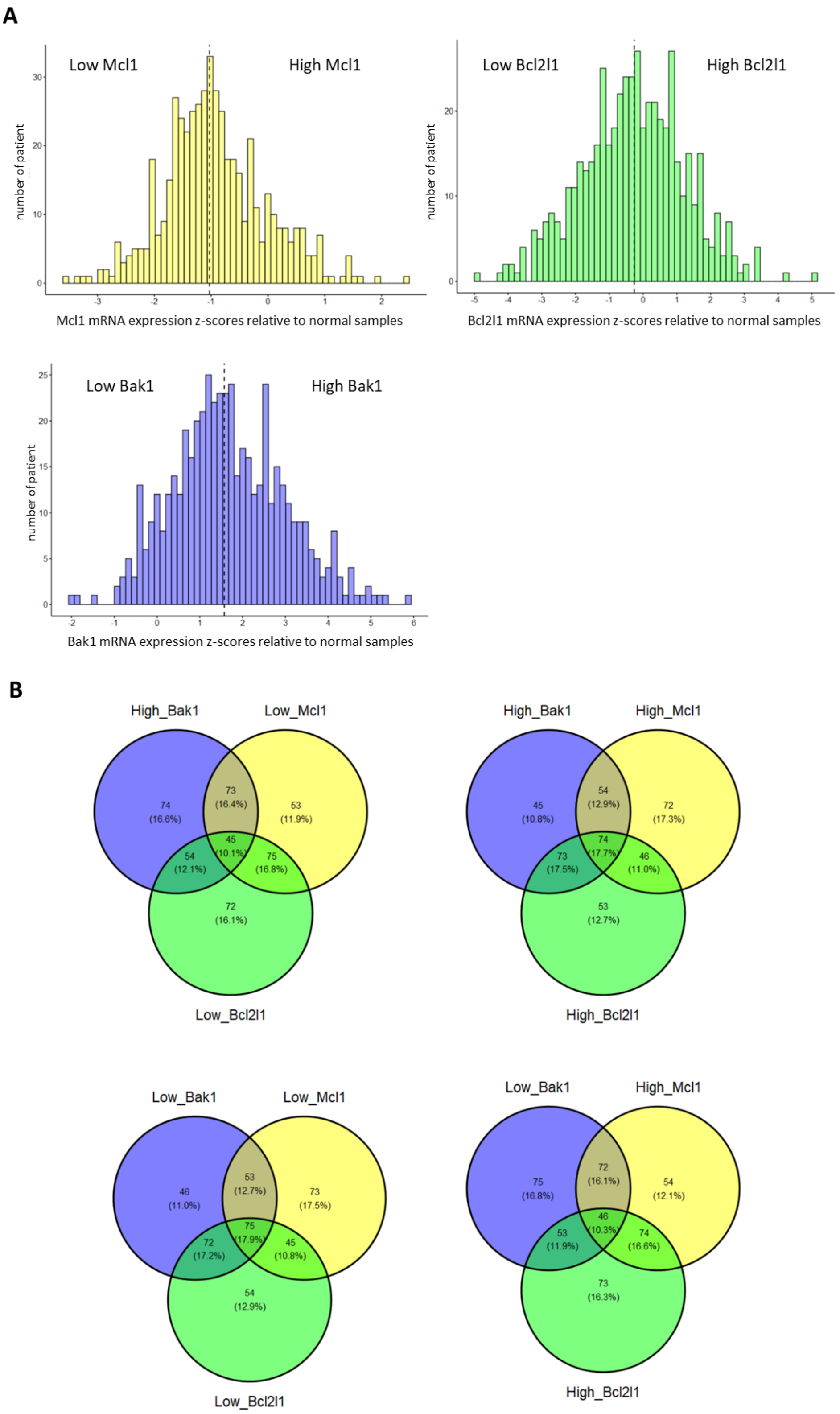

Figure S9. Subsets of LUAD patients with high/low Bak and high/low Bcl-xL and Mcl-1 levels. (**A**) The histogram of Mcl1, Bak1, Bcl2l1 mRNA expression z-scores relative to normal samples (log RNA Seq V2 RSEM). The vertical dashed line shows the median of z-score distribution that divide the patients by two groups (**B**) The Venn diagrams representing the four groups of patients (High

Bak1 & Low Mcl1 & Low Bcl2l1; Low Bak1 & Low Mcl1 & Low Bcl2l1; High Bak1 & High Mcl1 & High Bcl2l1; Low Bak1 & High Mcl1 & High Bcl2l1) that was used for survival analysis.
